# Drainage for complete obstruction of the posterior bile duct after pancreatoduodenectomy with a forward-viewing echoendoscope

**DOI:** 10.1055/a-2599-6843

**Published:** 2025-05-28

**Authors:** Shin Yagi, Susumu Hijioka, Yoshikuni Nagashio, Shota Harai, Mark Chatto, Yutaka Saito, Takuji Okusaka

**Affiliations:** 1 13874 Department of Hepatobiliary and Pancreatic Oncology, National Cancer Center Japan, Tokyo, Japan; 2 37571 Department of Medicine, Makati Medical Center, Manila, Philippines; 3 13874 Endoscopy Division, National Cancer Center Japan, Tokyo, Japan

The first-line treatment for hepaticojejunostomy anastomotic stenosis is balloon enteroscopy-assisted endoscopic retrograde cholangiopancreatography, which is difficult to perform in some cases 1 , despite the usefulness of a salvage method using a forward-viewing (FV) echoendoscope 2 3 . Here, we report a case of endoscopic ultrasound-guided biliary drainage (EUS-BD) using am FV echoendoscope in a patient with complete obstruction of the right posterior hepatic duct after surgery.

A 50-year-old man underwent a pancreaticoduodenectomy for pancreatic head cancer. The patient presented with an infraportal anomaly in the right posterior bile duct ( Fig. 1 **a, b** ) 4 . Ignorance of this anomaly during a pancreaticoduodenectomy led to complete iatrogenic transection of the right posterior hepatic duct, leading to its complete obstruction and resulting in postoperative cholangitis development ( Fig. 2 **a–c** ). Percutaneous transhepatic biliary drainage (PTBD) was performed for cholangitis ( Fig. 2 **d, e** ); however, the guidewire was unable to advance from the PTBD side to the intestine owing to complete obstruction. Therefore, we decided to perform EUS-BD with an FV echoendoscope ( Video 1 ). An FV echoendoscope was inserted to visualize the complete obstruction near the hepaticojejunostomy anastomosis ( Fig. 3 **a–c** ). A 19G needle (EZ Shot 3 Plus; Olympus Medical Systems, Tokyo, Japan) was used to puncture the right posterior hepatic duct, and a 0.025-inch guidewire was inserted into the bile duct ( Fig. 4 **a, b** ). The fistula was dilated with a 6-mm balloon catheter (REN; Kaneka Medix Corp., Osaka, Japan), and a fully covered metal stent (BONASTENT M-Intraductal, 8 mm, 3 cm; Medicoʼs Hirata, Tokyo, Japan) was placed ( Fig. 4 **c–e** ). After stent placement, a good outflow of contrast from the PTBD to the intestinal side was observed. A few days later, the PTBD was removed ( Fig. 5 **a–c** ); no recurrence of cholangitis was observed, and the patient was discharged.

**Fig. 1:**
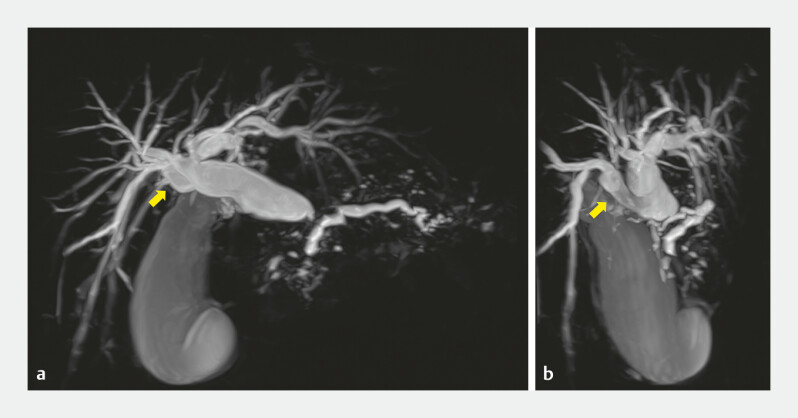
Preoperative magnetic resonance imaging showed an infraportal anomaly of the right posterior bile duct (arrow).

**Fig. 2:**
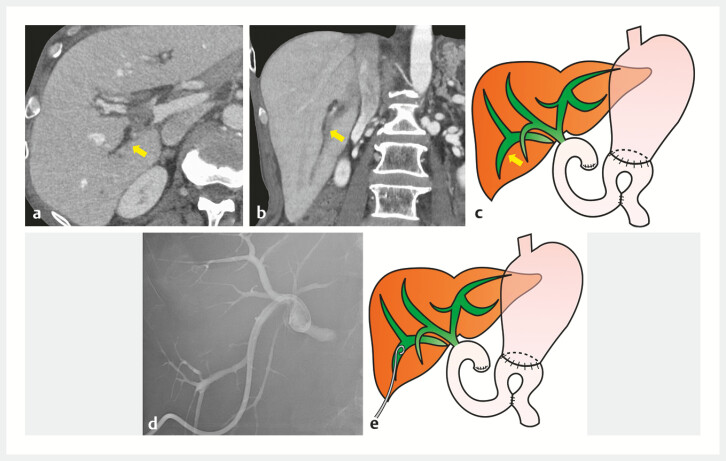
**a–c** Computed tomography scan images and schema showed the completely obstructed and dilated right posterior hepatic duct (arrow). **a, b** Axial and coronal images. **d, e** Radiographic image and schema showed the right posterior hepatic duct after percutaneous transhepatic biliary drainage placement.

Endoscopic ultrasound-guided biliary drainage using a forward-viewing echoendoscope in a patient with complete obstruction of the right posterior hepatic duct after pancreaticoduodenectomy.Video 1

**Fig. 3:**
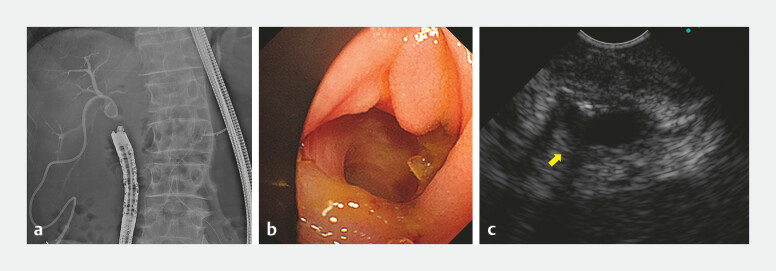
**a** Radiographic image showed the forward-viewing echoendoscope inserted near the hepaticojejunostomy anastomosis. **b** Endoscopic image showed the hepaticojejunostomy anastomosis. **c** Endoscopic ultrasound image showed the right posterior hepatic duct (arrow) visualized near the hepaticojejunostomy anastomosis.

**Fig. 4:**
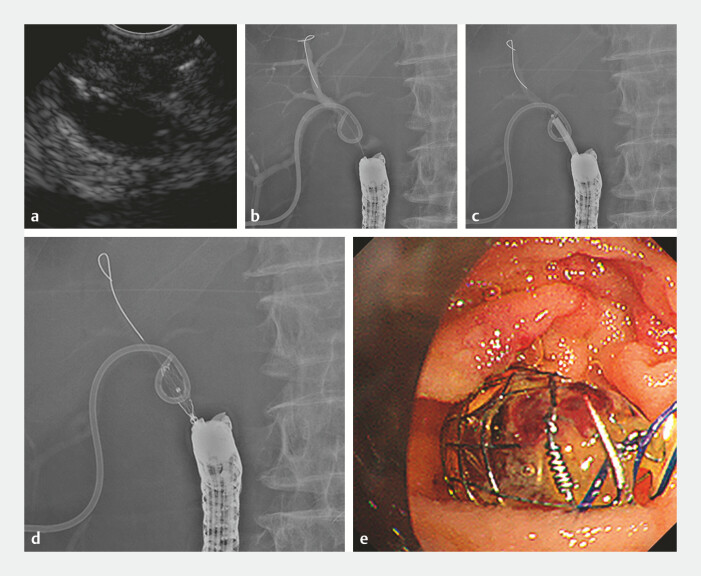
**a** Endoscopic ultrasound image showed puncture of the right posterior hepatic duct using a forward-viewing echoendoscope. **b** Radiographic image showed the insertion of a guidewire into the bile duct. **c** Radiographic image showed dilation of the fistula with a 6-mm balloon catheter. **d, e** Radiographic image and endoscopic image after the placement of a fully covered metal stent.

**Fig. 5:**
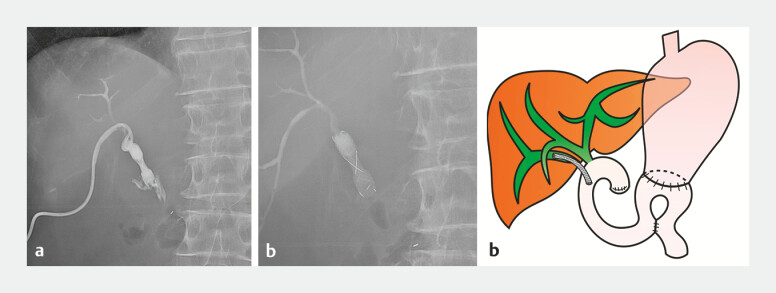
**a** Radiographic image showed contrast flow from the percutaneous transhepatic biliary drainage to the intestinal side. **b, c** Radiographic image and schema after percutaneous transhepatic biliary drainage removal.

Hence, EUS-BD with an FV echoendoscope may be a useful option for complete biliary obstruction caused by surgical procedures, as in the present case.

Endoscopy_UCTN_Code_TTT_1AS_2AH

## Notes

Endoscopy E-Videos https://eref.thieme.de/e-videos
*E-Videos* is an open access online section of the journal *Endoscopy* , reporting on interesting cases and new techniques in gastroenterological endoscopy. All papers include a high-quality video and are published with a Creative Commons CC-BY license. Endoscopy E-Videos qualify for HINARI discounts and waivers and eligibility is automatically checked during the submission process. We grant 100% waivers to articles whose corresponding authors are based in Group A countries and 50% waivers to those who are based in Group B countries as classified by Research4Life (see: https://www.research4life.org/access/eligibility/ ). This section has its own submission website at https://mc.manuscriptcentral.com/e-videos .
